# Modeling Agricultural Watersheds with the Soil and Water Assessment Tool (SWAT): Calibration and Validation with a Novel Procedure for Spatially Explicit HRUs

**DOI:** 10.1007/s00267-015-0636-4

**Published:** 2015-11-30

**Authors:** Awoke Dagnew Teshager, Philip W Gassman, Silvia Secchi, Justin T Schoof, Girmaye Misgna

**Affiliations:** Environmental Resources and Policy, Southern Illinois University Carbondale, 1400 Douglas Dr, Carbondale, IL 62901 USA; Department of Economics, Center for Agricultural and Rural Development, Iowa State University, 560A Heady Hall, Ames, IA 50011 USA; Department of Geography and Environmental Resources, Southern Illinois University Carbondale, Faner Hall, Carbondale, IL 62901 USA; Department of Geography and Environmental Resources, Southern Illinois University Carbondale, 4537 Faner Hall, Carbondale, IL 62901 USA

**Keywords:** Watershed modeling, SWAT, Calibration, Flow, Landuse, HRUs

## Abstract

Applications of the Soil and Water Assessment Tool (SWAT) model typically involve delineation of a watershed into subwatersheds/subbasins that are then further subdivided into hydrologic response units (HRUs) which are homogeneous areas of aggregated soil, landuse, and slope and are the smallest modeling units used within the model. In a given standard SWAT application, multiple potential HRUs (farm fields) in a subbasin are usually aggregated into a single HRU feature. In other words, the standard version of the model combines multiple potential HRUs (farm fields) with the same landuse/landcover, soil, and slope, but located at different places of a subbasin (spatially non-unique), and considers them as one HRU. In this study, ArcGIS pre-processing procedures were developed to spatially define a one-to-one match between farm fields and HRUs (spatially unique HRUs) within a subbasin prior to SWAT simulations to facilitate input processing, input/output mapping, and further analysis at the individual farm field level. Model input data such as landuse/landcover (LULC), soil, crop rotation, and other management data were processed through these HRUs. The SWAT model was then calibrated/validated for Raccoon River watershed in Iowa for 2002–2010 and Big Creek River watershed in Illinois for 2000–2003. SWAT was able to replicate annual, monthly, and daily streamflow, as well as sediment, nitrate and mineral phosphorous within recommended accuracy in most cases. The one-to-one match between farm fields and HRUs created and used in this study is a first step in performing LULC change, climate change impact, and other analyses in a more spatially explicit manner.

## Introduction

Watershed heterogeneity and limitations associated with monitoring make it impractical to measure every aspect of the hydrological system (Pechlivanidis et al. 2011). Hydrological models are frequently used to overcome these limitations and extrapolate information from available measurements in both time and space to the watershed scale. Ecohydrological models are important for a wide range of applications such as water resources planning, watershed development and management, flood prediction and design, water quality evaluation, hydro-ecology, and climate change analyses. The Soil and Water Assessment Tool (SWAT) watershed-scale ecohydrological model (Arnold et al. 1998; Arnold and Fohrer 2005; Gassman et al. 2007) is currently one of the most widely used ecohydrological models and it has been extensively tested for a wide variety of watershed scales and environmental conditions worldwide (Gassman et al. 2007, 2014; Douglas-Mankin et al. 2010; Tuppad et al. 2011; Krysanova and White 2015; Bressiani et al. 2015).

Applications of SWAT typically involve delineation of a watershed into subwatersheds/subbasins that are then further subdivided into hydrologic response units (HRUs). HRUs are homogeneous areas of aggregated landuse, soil, and slope and are the smallest modeling units used in the model. The incorporation of HRUs in SWAT has provided flexibility for simulating a broad spectrum of conditions and supports adaptation of the model for watershed scales ranging from small field plots to entire river basins (Gassman et al. 2007).

ArcSWAT, the standard ArcGIS input interface for SWAT, can spatially identify potential HRUs within a subbasin based on the above-mentioned HRU definition principles. However, ArcSWT-generated HRUs do not necessarily correspond spatially to individual farm fields in a given standard SWAT application. In other words, multiple potential HRUs (farm fields) with the same landuse/landcover, soil, and slope, but located at different places of a subbasin, are considered as one HRU. This characteristic of HRUs is referred, herein, as spatial non-uniqueness. Moreover, if lumping thresholds for HRU definition are used, spatial identification of individual HRUs will often be impractical. This is a key weakness in finding a one-to-one match between farm fields and HRUs, and hence assigning and presenting various inputs (e.g., rotation, tile drainage, manure and fertilizer application rates, etc.) and outputs at the HRU level. In this study, however, we introduce a data pre-processing procedure, discussed below, to create HRUs that are spatially unique so that there is a one-to-one match between HRUs and farm fields in a subbasin.

There have been other recent efforts in mapping SWAT outputs to a field level for identifying priority pollutant-contributing areas (e.g., Ghebremichael et al. 2010; Daggupati et al. 2011; Pai et al. 2012) so that appropriate management actions could be taken for specific land parcels. However, establishing spatially explicit (unique) HRUs prior to a SWAT simulation rather than after the model has been run could be extremely useful in certain applications. Arabi et al. (2008) subdivided the 7.3 km^2^ Smith Fry watershed in Indiana into 97 subbasins covering an average of 3 ha each, and used dominant landuse and soil type combinations such that each subbasin consisted of a single HRU, in order to more accurately simulate the impacts of different best management practices (BMPs) on sediment loss. Bekele and Nicklow (2005) used a similar delineation scheme for the 133 km^2^ Big Creek watershed in southern Illinois to assess the role of agricultural landscapes in reducing non-point source pollution while maximizing annual gross margin earned through agricultural production. However, the average subbasin size they used (175 ha) was much larger than those used by Arabi et al. (2008). The use of dominant HRUs (i.e., subbasins equivalent to HRUs) is not practical for many SWAT applications that are conducted at larger scales because key landscape, soil, and other details cannot be accounted for at the level of accuracy needed for the type of analyses being performed.

The ability to defining spatially unique HRUs in delineated subbasins prior to a SWAT simulation could be useful in simplifying assignment of inputs (rotation, tile drainage, manure application, etc.) and mapping of inputs/outputs at HRU level. It is important to note though that such “spatially defined HRUs” in the standard SWAT interfaces are still not simulated in a spatial manner within a simulation. A similar study on spatial one-to-one correspondence between HRUs and fields was done recently by Kalcic et al. (2015) for a small watershed (56 km^2^) in west-central Indiana. They used the Common Land Unit (CLU) field boundary data developed by the U.S. Department of Agriculture along with assigned unique soil names to create spatially unique HRUs in a subbasin. After clean-up of the CLU boundaries using ArcGIS and updates of the soil database for the new unique soil names, ArcSWAT was used to configure the model. Kalcic et al.'s work was successful in demonstrating how ArcSWAT could be utilized to create a one-to-one correspondence between HRUs and CLU fields. However, one must purchase the CLU data, distributed prior to the 2008 Farm Bill, to utilize their approach, and their methodology is of course not replicable for watersheds for which shape files of field boundaries are not available. Our procedure extends this previous work by relying only on raster data, which is generally freely available.

The SWAT model also requires many input parameters related to landuse, soil, weather, topography, water quantity and quality, which may need to be calibrated and validated prior to using the model for specific analyses. Calibration and validation of a SWAT model for a watershed is essential for reducing uncertainties and increasing the confidence of the user for effective and efficient analysis (White and Chaubey 2005; Jha 2011). SWAT can be calibrated and validated at the daily, monthly or annual time scales depending on the purpose of the specific modeling exercise. The most commonly calibrated SWAT output is streamflow, especially at annual and monthly time steps, although an increasing number of SWAT studies are reporting testing of daily streamflow results (Arnold et al. 2012; Gassman et al. 2007, 2014; Douglas-Mankin et al. 2010; Tuppad et al. 2011; Bressiani et al. 2015). Streamflow is calibrated more often than water quality in part because it is essential for the other water quality components of the model (Gikas et al. 2005) and also because observed flow data are relatively abundant. On the other hand, sediment and nutrient parameters are not calibrated and validated as often, especially at the daily time scale (Arnold et al. 2012; Gassman et al. 2007, 2014; Douglas-Mankin et al. 2010; Tuppad et al. 2011; Bressiani et al. 2015). Calibration and validation of water quality parameters (sediment and nutrients) of SWAT at coarser time scales is mainly attributed to scarcity of observed water quality data at finer time scales (Wu and Chen 2009).

For water quantity and quality analysis in SWAT, there are three groups of parameters: Flow, Sediment, and Nutrients, which could be calibrated either separately (e.g., Muleta and Nicklow 2005; Gikas et al. 2005; Jha et al. 2007; Chahinian et al. 2011, etc.) or simultaneously (Kaur et al. 2004; Tolson and Shoemaker 2008; Wu and Chen 2009, etc.). Though challenging, the latter procedure seems to be preferable for improved calibration and validation results (Chahinian et al. 2011) due to the fact that certain parameters, such as the curve number at moisture condition II (CN2), affect all flow, sediment, and nutrient concentrations. Proliferation of computers with high processing capacity is likely to lead to greater use of simultaneous calibration. In this study, we primarily perform simultaneous calibration/validation and also perform separate calibration/validation procedures for comparative purposes.

The objectives of this study are, therefore, to (1) present an ArcGIS-based NASS Cropland Data Layer (CDL) (NASS 2012) landuse/landcover data and soil data pre-processing method to create spatially unique HRUs in a subbasin, (2) show the significance of the pre-processing procedure in assigning and visualizing inputs and/or outputs, and providing a suitable platform for future landuse change impact analysis at the farm field level, and (3) utilize the results of the pre-processing procedure during ArcSWAT configuration and calibration/validation of the SWAT model for two Midwestern U.S. watersheds at daily, monthly, and annual time scales.

## Materials and Methods

### Study Area

**Fig. 1:**
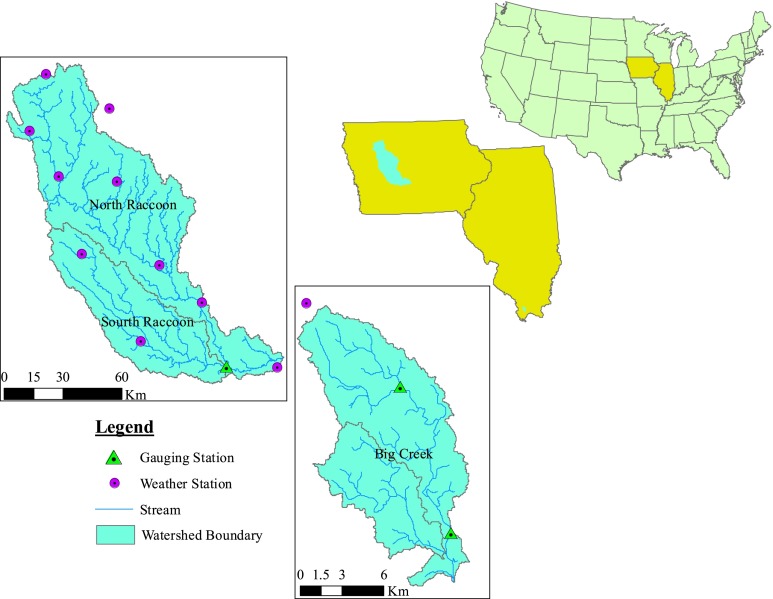
Study area and locations of weather, flow, sediment, and nutrient observation stations

Based on availability of data and previous research, two watersheds in the midwestern U.S. have been selected for this study (Fig. 1): (1) the Big Creek River watershed (BRW) in Illinois, which is a combination of two HUC12 or 12-digit watersheds (USGS (U.S. Geological Survey) 2012), and (2) the Raccoon River watershed (RRW) in Iowa, which is a combination of two HUC8 or “8-digit” watersheds (USGS (U.S. Geological Survey) 2012). Basic characteristics of each watershed are shown in Table 1.

**Table 1:** Main characteristics of the Big Creek and Raccoon River watersheds

Watershed name	Location (state)	Total area (km^2^)	Cropland area^a^ (% of total area)	MAT (°C)	MAP (mm)	Predominant crops and land use
Raccoon River	Iowa	9394	76.2	8.6	862	CORN, SOYB
Big Creek	Illinois	133	25.2	15	1095	PAST, FRST, SOYB

*MAT* mean annual temperature, *MAP* mean annual precipitation, *SOYB* soybeans, *FRST* forest, *PAST* pasture

^a^Cropland areas are average estimates of available USDA—NASS Cropland Data Layer from 2000 to 2010 (http://nassgeodata.gmu.edu/CropScape/). Moreover, cropland areas include fallow/idle croplands

The RRW is intensively agricultural (primarily corn and soybean), with agricultural land cover accounting for 76.2 % of the watershed. The BRW agricultural area, which is most frequently cropped in soybean, covers only 25.2 % of the watershed area. Both watersheds receive ample annual rainfall and therefore irrigation is not a common practice.

### Models

#### SWAT Ecohydrological Model

SWAT is a watershed-scale model developed to estimate the impacts of various landuse and management practices on water quantity and water quality over a continuous long period of time (Gassman et al. 2007). The model is proven to be efficient in using readily available data and in studying long-term impacts (Neitsch et al. 2011; Arnold et al. 2012). The model is usually executed at the daily temporal scale, although sub-daily time step applications can also be done. ArcSWAT (Olivera et al. 2006; SWAT 2015) and MWSWAT (MapWindow SWAT; George and Leon 2008) are the two GIS-based graphical input interfaces which could be used to configure a SWAT model in a GIS environment, which are commercial versus open source software, respectively.

#### SUFI-2 (Sequential Uncertainty Fitting Version 2)

The SUFI-2 calibration tool is part of the stand-alone, public domain SWAT Calibration and Uncertainty Program software (SWAT-CUP), which has its own interface and is used for sensitivity analysis, uncertainty analysis, and calibration and validation of SWAT model parameters (Abbaspour 2012). SUFI-2 is widely used (e.g., Abbaspour et al. 2007; Schuol et al. 2008; Faramarzi et al. 2009; Gong et al. 2011) mainly due to the relatively fewer required number of runs to reach an acceptable calibration results. SUFI-2 requires 2–30 times fewer runs than the other programs of the SWAT-CUP (Yang et al. 2008). In this study, therefore, SUFI-2 has been used for sensitivity analysis, and calibration and validation of the SWAT models.

Uncertainties from driving variables (e.g., rainfall), model, parameters, and observed data (e.g., flow) are all accounted for in parameter uncertainty during a SUFI-2 calibration/uncertainty analysis. The measure of the degree to which these uncertainties are accounted for is defined as the *P*-*factor* (the percentage of measure of data bracketed by the 95 % prediction uncertainty, 95PPU) and the *R*-*factor* (the ratio of average thickness of the 95PPU band to the standard deviation of the measured data). These two measures work together to bracket most of the measured data with the smallest possible uncertainty band. The regression correlation coefficient (*R*^2^) and the Nash–Sutcliffe coefficient of efficiency (NS_e_) between observed data and the final best simulation output are further quantifications of goodness of fit during SUFI-2 calibration/uncertainty procedure. While *R*^2^ (ranges from 0 to 1) measures how well the observed data are correlated to simulated values, NS_e_ (ranges from −∞ to 1) measures how well the observed and simulated data match. For both statistics, values of close to one indicate a well-calibrated and validated model. See Krause et al. (2005) for further description of the *R*^2^ and NS_e_, as well as statistical implications of using the two statistics for hydrologic and water quality model evaluations.

#### Land Use and Land Cover (LULC) and Soil Pre-processing Procedure

As discussed in “Introduction” section, HRUs are not spatially unique in a given standard SWAT application. Even though ArcSWAT can identify potential HRUs spatially, these HRUs are not spatially unique within a subbasin for a one-to-one match between agricultural fields and HRUs. Hence, an ArcGIS-based data processing methodology has been developed to overcome this problem before using ArcSWAT to create HRUs. Here, ArcGIS tools have been utilized to develop a landuse/landcover (LULC) and soil pre-processing procedure to define spatially unique HRUs with in a subbasin. These HRUs are still simulated in a nonspatial manner in SWAT but the soil, landuse, and topographic data used to define the HRUs are geo-referenced, which allows more realistic spatial identification of the model inputs and outputs, and easier linkages with other spatially explicit models such as economic or ecological ones.

This procedure requires freely available landuse and soil raster data, river networks, road networks, and pre-defined subbasins for the watershed to be processed. The general procedure is as follows:

1. Convert LULC to polygons (based on LULC type)
2. Exclude roads and “very small” urban lands from LULC polygons and re-assign them with the neighboring LULC type. In this procedure, roads are considered to be dividing lines between LULC types. Hence, this step helps to eliminate “unnecessary and small” polygons due to roads, very small towns, and isolated buildings in the middle of farmland or forest.
3. Split new LULC polygons with road and river networks. In addition to roads, river networks are also considered to be dividing lines between LULC types. Like roads, LULC types on opposite sides of river networks could potentially be different. However, in this particular procedure, forests and urban areas are considered intact throughout the analysis; hence, forest and urban area polygons are excluded from the split.
4. Eliminate “small” polygons. Here, small may be subjective but generally the size of the polygons eliminated depends on the size and LULC pattern of the watershed. Moreover, elimination has to be made so that the number of polygons created is less than the number of HRUs that could be managed by the SWAT simulation processing facilities. The result of this step can be exported to GoogleEarth and compared with the actual boundaries of fields for an accuracy check.
5. Intersect the final polygons, after elimination, with pre-defined subbasin polygons (from ArcSWAT). One must ensure that the subbasins used here are exactly the same as those created during the ArcSWAT watershed delineation.
6. Re-label LULC polygon codes by assigning different codes for the same LULC types within a subbasin. For example, if there are three CORN polygons within subbasin 1, then these polygons are re-assigned with names like COR1, COR2, and COR3. These same new crop types should also be added into the SWAT crop database but should all have the same crop parameters, i.e., crop parameters for CORN. The same is true for other LULC types.
7. Finally, convert the re-labeled LULC polygons into raster-based data which will be used for the actual analyses.
8. Use the updated LULC polygons to assign a dominant soil for each polygon for the watershed analyses.

**Fig. 2:**
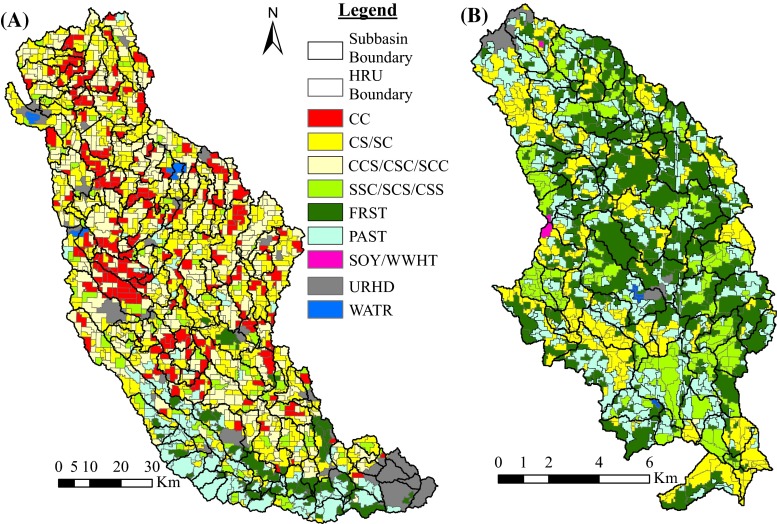
Crop rotations constructed for **a** RRW and **b** BRW (*C* corn, *S SOY* soybeans, *CC* continuous corn, *WWHT* winter wheat, *FRST* mixed forest, *PAST* pasture, *URHD* urban high density (developed), *WATR* open water body)

**Fig. 3:**
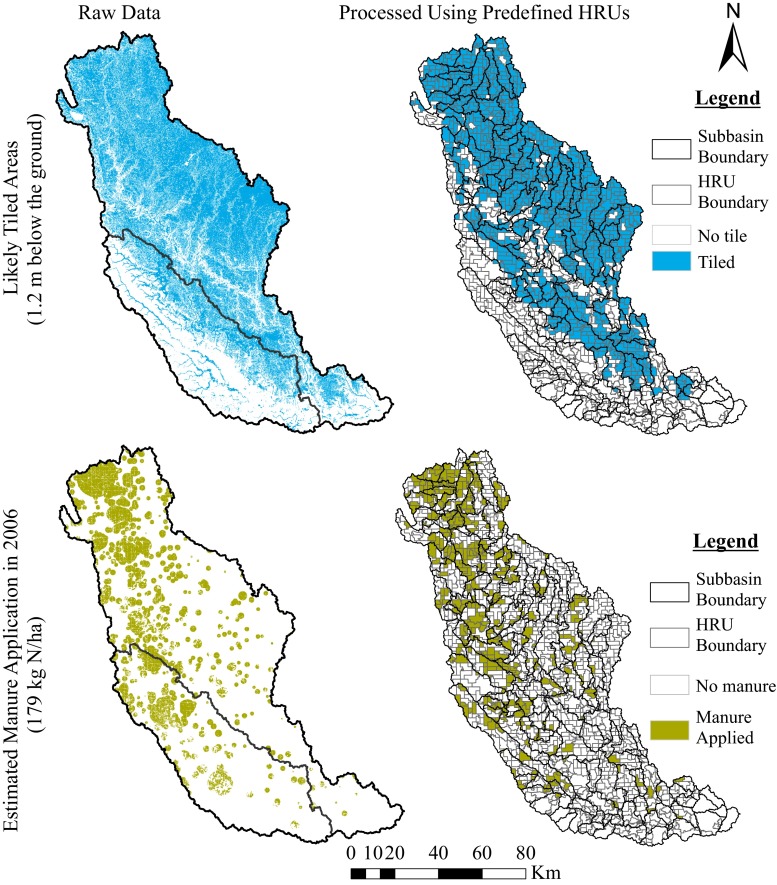
Distribution of tile drainage and manure applications in the Raccoon River watershed. (*Source* Iowa DNR website, http://www.igsb.uiowa.edu/nrgislibx/)

The main objective of the above procedure is to define spatially unique HRUs within a subbasin using ArGIS tools before an ArcSWAT model is developed. This will assist in assigning management input data in a spatially explicit manner for each HRU (Figs. 2 and 3), presenting output results at the HRU level, and assisting in explicitly analyzing future LULC change possibilities at the HRU level in SWAT modeling (but keeping in mind the limitation that the HRUs are not simulated spatially internally in SWAT).

**Fig. 4:**
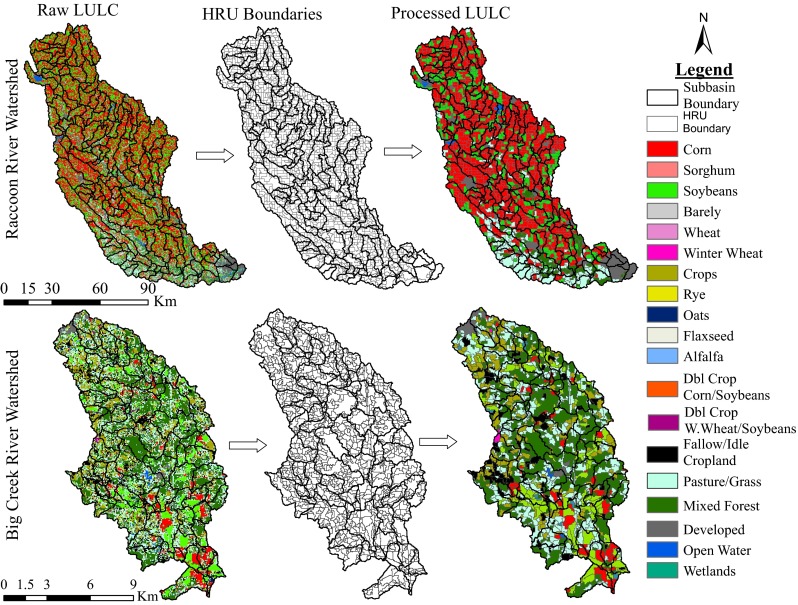
LULC pre-processing results for Big Creek and Raccoon River watersheds

The raw LULC layers, 2000 LULC for BRW and 2010 LULC for RRW, were utilized to define the HRU boundaries from which the new gridded LULC data were constructed (Fig. 4). Once the HRU boundaries (polygons) were set using a single year of data, the polygons were utilized in determining the dominant LULC type (i.e., cropland or non-cropland) for each polygon (HRU). Crop rotations (Fig. 2) for the cropland HRUs were then determined by overlaying multiple years of crop land use data on each polygon, using crop data obtained from the USDA Cropland Data Layer (CDL; USDA-NASS, 2012) for the Raccoon (2002–2010) and Big Creek (2000–2003) watersheds. Using multiple crop years to construct the land use is crucial in watersheds where farmers rotate annual crops, because the various crops can have very different water quality impacts, and using only 1 year of data may mask long-term trends in land cover and affect the accuracy of the calibration process.

#### Load Estimator (LOADEST)

The Load Estimator (LOADEST) is a software package developed by USGS to generate water quality parameters through regression based on observed water quality data (grab samples) and their corresponding flow rate and time of observation (Runkel et al. 2004). LOADEST has eleven pre-defined regression models to choose from. Moreover, the user-defined option of LOADEST can be utilized to incorporate additional parameters to improve the prediction capability of the regression equations. The regression model can be developed for daily, monthly, or annual data generation. In this study, LOADEST has been utilized to interpolate water quality data to be used during calibration and validation procedures.

### Data

Recent 30-m gridded Digital Elevation Model (DEM) data were downloaded from the United States Geological Survey (USGS) National Map Viewer and Download platform (USGS (U.S. Geological Survey) 2012). Likewise, 30-m gridded landuse (CDL; USDA-NASS, 2012) and county-based soil data from the Soil Survey Geographic Database (SSURGO) (USDA-NRCS, 2012) were downloaded from the USDA respective geospatial data gateways. Climate data (daily precipitation and maximum and minimum temperature) from the National Oceanic and Atmospheric Administration’s (NOAA) National Climatic Data Center (NCDC) (NOAA–NCDC, 2012) were obtained for ten weather stations for the RRW and one weather station for the BRW (Fig. 1).

Data were collected from one RRW stream gauge and two BRW stream gauges (Fig. 1) to perform the calibration/validation processes. While about 95 % of the RRW drains to the gauging station, about 64 and 17 % of the BRW area drains to its downstream and upstream gauging stations, respectively. Multiple sources were utilized in collecting daily flow and water quality (Total Suspended Sediment, TSS, Nitrate, NO_3_, Mineral Phosphorous, MINP) data, most of which were obtained from the Environmental Protection Agency STOrage and RETrieval (EPA-STORET) online database. Sources such as USGS, the Des Moines Water Works (DMWW), the Des Moines River Water Quality Network (DMRWQN), the Illinois Water Survey website, and individual e-mail requests were also utilized for flow and water quality data collection. The simulation periods for each watershed were determined based on the availability of water quality data as shown below (Table 2).

**Table 2:** Availability of observed water quality data and selected periods of simulations

Watershed Name	Sediment (assessment period)	Nitrogen (assessment period)	Phosphorous (assessment period)	Frequency of observed data values	Simulation period selected
Raccoon	1999–2010	1999–2010	1999–2010	5 per week to 1 per month	2002–2010
Big Creek	1999–2003	2000–2002	2000–2002	5 per week to 1 per month	2000–2003

For the watersheds considered in this study, urban, forest, and pasture landuse/landcover types were assumed to remain the same for the entire simulation periods during the calibration and validation processes. Muleta and Nicklow (2005) assumed that corn, soybean, pasture, and hay are grown without tillage in the BRW based on interviews with personnel from the Southern Illinois District office of the NRCS (Natural Resources Conservation Services). However, there is no evidence that manure applications occur or that tile drains are used in the BRW. In the RRW, on the other hand, information about tillage practices, manure applications, and tile drainage were available based on previous SWAT model studies reported by Jha et al. (2007, 2010) and Schilling et al. (2009). Jha et al. (2010) and Schilling et al. (2010) reported that approximately 77.5 % of the row cropland in Northern Raccoon watershed and 42 % in the South Raccoon watershed were tile drained (Fig. 3), and that tile drains were installed (1200 mm below the ground) on about 48 % of the cropland overall, based on data obtained from the Iowa Department of Natural Resources (IDNR 2013).

Estimated manure application distribution on cropland, at a rate of 160 lbs N/acre (~179 kg N/ha) from animal feeding operations in Iowa in 2006 (Fig. 3), was also obtained for the RRW from IDNR (2013). According to this data, about 20 % of the RRW area received manure in 2006. In this particular study, this rate (179 kg N/ha) was applied at the time of corn planting for each corn production season during the entire simulation.

Data for fertilizer application rates and tillage practices were estimated based on data collected from USDA-Economic Research Service (ERS) Agricultural Resources Management Survey (ARMS; USDA-ERS, 2013). State level estimates of fertilizer application rates and tillage practices for Iowa were available from ARMS for years 1997, 1998, 1999, 2000, 2001, 2005, and 2010. Data from the years within and closest to the simulation periods (2000, 2001, 2005, and 2010) were used for the RRW analysis. The average values of rates of nitrogen and phosphorous fertilizer applications, and percentages of three tillage practices [conventional (Cv), conservation (Cs), and no-till] for corn and soybeans were estimated for RRW using data from those years mentioned above (Table 3).

**Table 3:** Fertilizer/manure application rates and presence of tiles and tillage practices

Watershed name	Crop type	Rotation	Fertilizer		Manure (kg N/ha)	Tile	Tillage
			kg N/ha	kg P/ha			
Raccoon	CORN	CORN after CORN	165	65	179	Yes	No-till, Cs, Cv
	CORN	CORN after SOYB	150	70			
	SOYB	SOYB after CORN	15	55	0		
	SOYB	SOYB after SOYB	0	0			
Big Creek	CORN	Irrelevant	170	85	0	No	No-till
	SOYB	Irrelevant	0	68	0		

The BRW is located south of the main southern Illinois crop production region, and thus, basing the management practice on state average values could not be adequately justified. Therefore, the estimated nutrient application rates and tillage practices used for the BRW SWAT simulations were based on expert opinion provided by local agency personnel (Table 3).

### Model Baseline Setup

The SWAT2009/rev. 481 and ArcSWAT 2009.93.7b were utilized as a tool and interface, respectively, in this study. Pre-processed landuse and soil data along with the DEM data were used to construct subbasins and HRUs for each watershed simulation. Since landuse and soil data were already processed, threshold values of 0 % for landuse, 0 % for soil, and 0 % for slope were used during the HRU definition processes. Moreover, a single slope class was used to avoid HRU fragmentation and ensure that each HRU was matched to individual farm fields in a subbasin. Accordingly, 2481 HRUs in 154 subbasins and 1644 HRUs in 79 subbasins were constructed for the RRW and BRW, respectively. In addition to the simulation periods indicated in Table 2, 2-year warm-up periods were introduced for each watershed.

Crop rotations were constructed using multiple years of LULC data for both watersheds (Fig. 2). More than 40 % of the RRW watershed area was planted in either continuous corn or 2 corn years in a 3-year rotation (CC = 13.9 % and CCS/CSC/SCC = 30.0 %) for years 2002–2010. Two-year CS/SC rotations cover about 30.6 % and 2 years of soybean in 3-year rotations (SSC/SCS/CSS) cover about 5.8 % of the watershed. The remaining 20 % of land area in the watershed area was either developed, forested, managed as pasture or an open water body. For the BRW, CS/SC and SSC/SCS/CSS rotations account for about 21.6 and 16.4 % of the watershed area, respectively, for years 2000–2003. Forest (32.7 %) and pasture (27.5 %) account for the majority of the BRW area; the remaining 2 % of the watershed was either developed areas or open water body. The pre-processing procedure resulted in negligibly small percentage of rotations of soybeans followed by a year of double-cropped winter wheat and soybeans (SOY/Dbl crop WWHT-SOY) and 2 years of corn in 3-year rotations in the BRW (0.2 and 0.1 %, respectively). Since all of the above-mentioned rotation percentage values were results of estimations based on the pre-processing procedure, the proportion of agricultural/pasture/forest/developed/open water areas could be slightly different from what was reported in Table 1 or the raw LULC type proportions of the base year used for pre-processing (see “LULC and Soil Pre-processing Procedure” section).

Management operations such as fertilizer and/or manure applications, tillage practices, and tile drainage were implemented for each pre-defined HRU according to the availability of data for the watershed. For the RRW, tile drainage and manure applications were distributed throughout HRUs based on the acreage of the management operation in each HRU. If an operation (tile drainage or manure application) covered more than 50 % of HRU’s area, then the operation was applied for the HRU. Otherwise, the operation was excluded. Figure 3 shows original tile drainage and manure application distribution data along with processed distribution results used in this study for the RRW. Fertilizer application rates estimated based on data from the USDA-ERS were applied for each HRU in accordance with crop rotations (Table 3).

### SWAT Calibration and Validation

Both automatic calibration (using SUFI-2) and manual calibration procedures were used in this study. Initially, the curve number at moisture condition II (CN2), saturated hydraulic conductivity of the soil (SOL_K), and available water capacity of the soil (SOL_AWC) values for various LULC types were adjusted manually to calibrate, at least partially, the annual water/sediment/nutrient balances and the baseflow contributions to the average annual total water yields of both watersheds. In addition to the above parameters, the threshold depth of water in the shallow aquifer required for return flow to occur (GWQMN) for BRW, and fraction of porosity from which anions are excluded (ANION_EXCL) for the RRW were found to be sensitive to annual water yield and its baseflow fraction, and annual nitrate output, respectively, and hence were adjusted manually as well.

Previous studies on RRW show that baseflow is an important component of the water balance for the watershed (Jha et al. 2007, 2010; Schilling et al. 2009). Hence, baseflow needs to be calibrated before stream flow and other components to enhance modeling accuracy. The automated method of estimating baseflow, developed by Arnold and Allen (1999), is used to estimate the fraction of baseflow contribution for the total annual flow. Accordingly, for the RRW, baseflow is estimated to contribute about 61.5 % on an average annual basis for 2002–2010. This value is consistent with Jha et al. (2007) estimates of 58 % for 1981–2003 using the same method of estimation. Similar estimates for the BRW resulted in baseflow contributions of about 28 % on an average annual basis for 2000–2003. Since previous studies on the BRW did not consider baseflow separation during calibration/validation of the watershed, there was no information to compare the result to. The BRW baseflow component is expected to be lower than the RRW due to the lack of tile drainage in the watershed. However, the BRW LULC is mainly forest and pasture which would increase the baseflow contribution. Therefore, it is possible that the baseflow contribution for BRW may be underestimated due to the short duration (4 years) of flow record considered in this study.

The calibration/validation periods for RRW and BRW were 2002–2007/2008–2010 and 2000–2001/2002–2003, respectively, except that the validation period for the BRW nutrient simulation was only the year 2000 due to data limitations. The upstream gauging station of the BRW was used as further verification of the model performance for the watershed. Both simultaneous and separate calibration/validation procedures were performed by changing relevant SWAT parameters (Appendix Table 6). These calibration and validation processes were performed at daily and monthly time steps for each watershed, and also at an annual time step for the RRW. The model predictions, during both calibration and validation periods, were evaluated using graphical and statistical comparisons. The *R*^2^ and NS_e_, statistical measures of SUFI-2 discussed in “SUFI-2 (Sequential Uncertainty Fitting version 2)” section, were used for evaluation.

Evaluations were performed at daily, monthly, and annual time scales. Though there are no firm criteria for minimum required values of *R*^2^ and NS_e_, an NS_e_ value of at least 0.5 is recommended by Moriasi et al. (2007) for the SWAT model to be satisfactory for hydrologic and pollutant loss evaluations on a monthly time scale. Therefore, Moriasi’s recommendation was considered as the minimum goal for both *R*^2^ and NS_e_ at monthly time scales, and the minimum values of *R*^2^ and NS_e_ for daily and annual time scales were expected to be less and greater than that of Moriasi’s recommendation, respectively.

## Results and Discussion

### LULC and Soil Pre-processing Procedure

The percentage of each LULC type changed as a result of the pre-processing procedure (Fig. 4). Decreases from 11.5 to 10.0, 7.3 to 5.9, and 0.7 to 0.5 % for pasture, developed, and open water areas, respectively, and increases from 75.6 to 79.2 and 4.1 to 4.4 % of agricultural and mixed forest areas, respectively, were observed for the RRW. For the BRW, decreases from 43.2 to 37.7, 29.9 to 27.5, and 0.3 to 0.2 % for agricultural, pasture, and open water areas, respectively, and increases from 23.7 to 32.6 and 0.9 to 1.5 % for mixed forests and developed areas, respectively, were observed due to the pre-processing procedure.

Some of the LULC changes above may look objectionable at first glance; however, it should be noted these changes are similar to those that are inevitable if threshold levels are used in SWAT to eliminate minor LULCs during HRU definition. Detailed descriptions of the processes during the HRU definition in SWAT modeling can be found in the ArcSWAT user’s guide by Winchell et al. (2007). The data pre-processing procedure introduced in this study performs a similar function in defining the HRUs except that the pre-processed data helps define spatially unique HRUs in a subbasin during ArcSWAT configuration. It is also possible that the quality of the LULC data may have an influence on the results of the pre-processing procedure, though exploring this issue goes beyond the scope of this work, and therefore, we cannot offer firm conclusions on this issue. According to Johnson and Mueller (2010), “the overall quality and accuracy of the CDLs has steadily increased over time due to better ground truth and greater access to imagery.” Therefore, using a more recent (2010) LULC data for the RRW may be the reason for lower changes in LULC between raw and processed data, compared to the 2000 LULC data used for BRW that produced higher changes in LULC. However, this could also be due to the fact that the RRW landscape is much more homogenous than that of the BRW.

One of the applications of results of this procedure is that multiple years of LULC data could be utilized to determine HRU-level crop rotations explicitly (Fig. 2). This will in turn help determine annual fertilizer application rates more accurately as shown in Table 3. In addition to this, HRU boundaries from the pre-processing procedure were used to determine the soil type for each HRU. The dominant soil type for each HRU polygon was assigned, and the resulting soil data were used for the SWAT modeling. Pre-defined HRUs were further utilized to determine which area was likely tiled and received manure. Accordingly, processing raw tile drainage and manure application data from the IDNR through the pre-defined HRUs resulted in a tiled area of 57 % (as compared to the original 48 %) of the entire RRW and manure application in 20 % of the watershed, respectively.

### Calibration and Validation

The calibration process resulted in total annual average water yield estimates of ~260 and ~470 mm for the RRW and BRW, respectively. The baseflow fractions were simulated to be ~56 % for the RRW and ~31 % for the BRW. The value for the RRW was consistent with previous studies by Schilling et al. (2009), Jha et al. (2007), and Schilling and Zhang (2004) with estimated baseflow fractions of 56.5 % (1995–2004), 56 % (1981–2003), and 54 % (1972–2000), respectively. The average annual tile flow in the RRW was simulated to be ~55 mm (22 % of the average total annual water yield) in this study which was also consistent with 56 mm (1986–2004) estimated by Jha et al. (2010) and about 51 mm (1995–2004) estimated by Schilling et al. (2009). For the BRW, the simulated value of the baseflow fraction is consistent with the 28 % fraction obtained from using the automated method of baseflow separation mentioned in “SWAT Calibration and Validation” section.

**Fig. 5:**
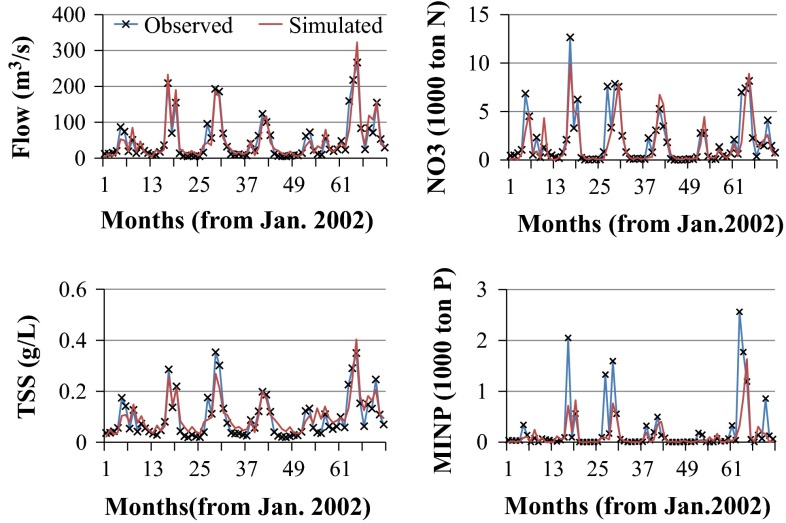
Time serious of simulated and observed monthly flow (*upper left*), suspended sediment (*lower left*), nitrate load (*upper right*), and mineral phosphorous load (*lower right*) data during calibration period (2002–2007) for RRW

Comparisons between observed and simulated flow, sediment, nitrate, and mineral phosphorous (Table 4; Fig. 5 and Appendix Figs. 9 and 10) show that the calibration processes produced representative SWAT models for both watersheds, with more accurate results for the RRW. In the RRW, annual and monthly comparisons (Table 4) were comparable with previous results by Jha et al. (2007; 2010), except the MINP values. However, at daily time scale, much better results of *R*^2^/NS_e_ were obtained in this study compared to the daily calibration results of Jha et al. (2010). The *R*^2^/NS_e_ values for daily calibration were 0.81/0.80 for flow, 0.54/0.53 for sediment concentration, 0.69/0.66 for NO_3_ load, and 0.47/0.45 for MINP load.

**Table 4:** SWAT model calibration and validation results for RRW and BRW at various time scales (simultaneous calibration procedure)

Watershed name	Calibrated output	Time step	Calibration		Validation	
			*R* ^2^	NS_e_	*R* ^2^	NS_e_
RRW	Flow	Daily	0.81	0.80	0.87	0.86
		Monthly	0.89	0.88	0.92	0.91
		Annual	0.97	0.95	–	–
	TSS	Daily	0.54	0.53	0.52	0.51
		Monthly	0.82	0.80	0.77	0.74
		Annual	0.93	0.91	–	–
	NO_3_	Daily	0.69	0.66	0.52	0.14
		Monthly	0.72	0.70	0.57	0.32
		Annual	0.86	0.80	–	–
	MINP	Daily	0.47	0.45	0.40	0.36
		Monthly	0.46	0.36	0.54	0.48
		Annual	0.96	0.72	–	–
BRW	Flow	Daily	0.64	0.63	0.72	0.68
		Monthly	0.66	0.54	0.77	0.56
	TSS	Daily	0.45	0.44	0.36	0.34
		Monthly	0.74	0.70	0.57	0.55
	NO_3_	Daily	0.31	0.11	0.00	−0.45
		Monthly	0.56	0.30	0.03	−1.40
	MINP	Daily	0.45	0.39	0.50	0.47
		Monthly	0.85	0.68	0.99	0.98

**Fig. 6:**
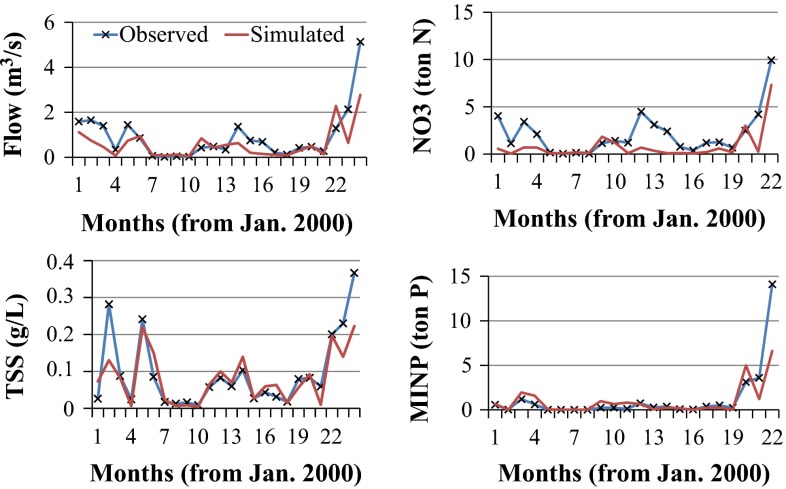
Time serious of simulated and observed monthly flow (*upper left*), suspended sediment (*lower left*), nitrate load (*upper right*), and mineral phosphorous load (*lower right*) data during calibration period (2000–2001) for BRW

Having a longer and more recent time series of data to calibrate and validate to, it was expected that the RRW would do much better during calibration and validation than the BRW. These expectations were proven accurate by the BRW calibration results (Table 4; Fig. 6 and Appendix Fig. 11). Generally, in the BRW, less accurate calibration results were obtained. Due to the very short series of data available for the BRW, annual calibration procedures using SUFI2 were not performed. Daily flow and sediment concentration calibration results were consistent with previous studies by Muleta and Nicklow (2005) and Bekele and Nicklow (2007) for the watershed. Moreover, the monthly calibrations (*R*^2^/NS_e_ value of 0.66/0.54 for flow, 0.74/0.70 for sediment concentration, 0.56/0.30 for NO_3_ load, and 0.85/0.68 for MINP load), which were performed only in this study, resulted in significantly improved calibration values, especially during water quality calibration.

**Fig. 7:**
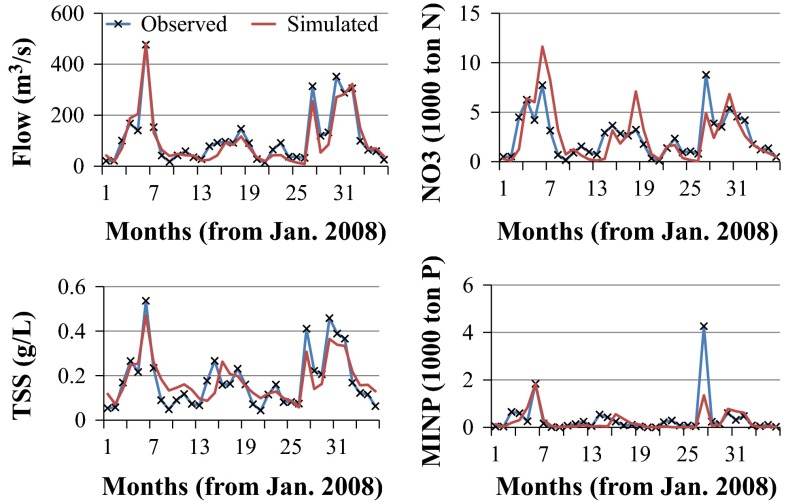
Time serious of simulated and observed monthly flow (*upper left*), suspended sediment (*lower left*), nitrate load (*upper right*), and mineral phosphorous load (*lower right*) data during validation period (2008–2010) for RRW

**Fig. 8:**
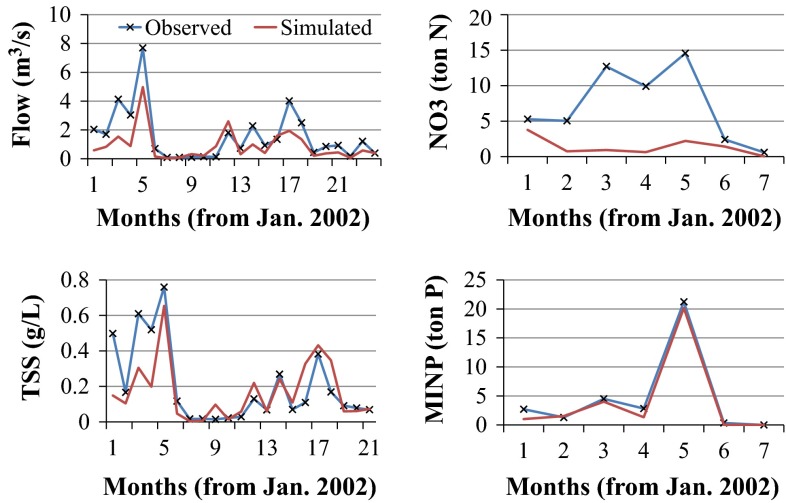
Time serious of simulated and observed monthly flow (*upper left*), suspended sediment (*lower left*), nitrate load (*upper right*), and mineral phosphorous load (*lower right*) data during validation period (2002–2003) for BRW

Validations of the RRW were performed for years 2008–2010 for all the calibrated outputs at the same gauging station that the calibration was performed for. Even though the *R*^2^/NS_e_ values during validation were generally less than the calibration period, most of the values were satisfactory (Table 4; Fig. 7 and Appendix Figs. 12, 13). Validations for BRW, on the other hand, were done for years 2002–2003 for flow and sediment, and for year 2002 for nitrate and mineral phosphorous at the downstream gauging station. The *R*^2^/NS_e_ values of flow and mineral phosphorous during the validation periods were better than the calibration period (Table 4; Fig. 8 and Appendix Fig. 14). The BRW model was also validated for flow at the upstream gauging station for years 2000–2003. The *R*^2^/NS_e_ values for the upstream gauging station were 0.45/0.42 at daily and 0.58/0.46 at monthly time steps.

Finally, comparisons were made between *R*^2^/NS_e_ results of simultaneous and separate (stepwise) calibration procedures. As can be deduced from the results presented in Table 5, neither procedure provided a clear advantage on the basis of the *R*^2^/NS_e_ values. Calibrated SWAT parameter values are presented in Appendix Table 7 in the appendices for convenience and future reference.

**Table 5:** Comparison of *R* ^2^ and NS_e_ results between simultaneous and separate calibration procedures for RRW and BRW

Watershed name	Calibrated output	Time step	Simultaneous calibration		Separate calibration	
			*R* ^2^	NS_e_	*R* ^2^	NS_e_
RRW	Flow	Daily	0.81	0.80	0.80	0.78
		Monthly	0.89	0.88	0.91	0.90
	TSS	Daily	0.54	0.53	0.55	0.55
		Monthly	0.82	0.80	0.88	0.88
	NO_3_	Daily	0.69	0.66	0.59	0.55
		Monthly	0.72	0.70	0.75	0.74
	MINP	Daily	0.47	0.45	0.47	0.44
		Monthly	0.46	0.36	0.47	0.46
BRW	Flow	Daily	0.64	0.63	0.68	0.65
	TSS	Daily	0.45	0.44	0.40	0.40
	NO_3_	Daily	0.31	0.11	0.17	0.08
	MINP	Daily	0.45	0.39	0.41	0.40

## Conclusions

The LULC pre-processing procedure introduced in this study was the first step in defining spatially unique HRUs for the SWAT model before the simulations. Its applications in assigning and presenting certain management data inputs and presenting model outputs at the HRU level in a spatially explicit manner including accounting for multi-year crop rotations were demonstrated in this study. The procedure could be used to predict LULC changes in a watershed at the HRU level in studies such as multi-objective management of ecosystem services (Bekele and Nicklow 2005), coupling agent-based LULC change modeling with SWAT model (Ng et al. 2011), and strategic targeting and prioritization of areas that need certain kind of management practice implementation (Daggupati et al. 2011). Moreover, the approach could be utilized anywhere in the world as long as landuse, soil, DEM, and road network data are available. In the absence of road network data, the procedure could still be used but the accuracy of the results will likely be lower.

The *R*^2^ and NS_e_ results (Table 4) for the RRW showed that the SWAT model was able to replicate annual, monthly, and daily streamflow, as well as sediment, nitrate, and mineral phosphorous, within recommended accuracy as suggested by Moriasi et al. (2007) except for a few cases. Due to limited and older observed data, less accurate results were obtained for the BRW simulations. However, except for nitrate, the BRW monthly calibration results were also satisfactory as per Moriasi’s performance ratings. Despite the comparability of both simultaneous and separate calibration procedures, the importance of simultaneous calibration with respect to its accuracy presented in this study should also be noted. It is also important to remember that there are bias issues with estimating pollutant loads with LOADEST, especially for daily load estimates (Stenback et al. 2011). Thus, future research is needed to improve upon current available load estimation methods.

Finally, the HRUs simulated in this study still do not overcome the problem that HRUs are simulated nonspatially within the actual SWAT simulations. An alternative approach is currently being developed for the SWAT model that allows spatial representation of different landscapes within a subbasin as well as simulated routing of flow and pollutants between the different landscapes (e.g., see Bosch et al. 2010; Arnold et al. 2010; Bonumá et al. 2014). HRUs are delineated within the landscape units in this approach; these HRUs are still not spatially identified, although a single HRU could be used to represent a given landscape unit depending on the application [Arnold (2014) Personal communication. USDA-ARS Grassland, Soil and Water Research Lab., Temple, TX]. Interfacing the HRU approach described in this study with the landscape structure being developed for SWAT will provide an even more enhanced method for spatially representing landuse, management practices, and other landscape-specific characteristics within the model.

## Figures and Tables

**Fig. 9:**
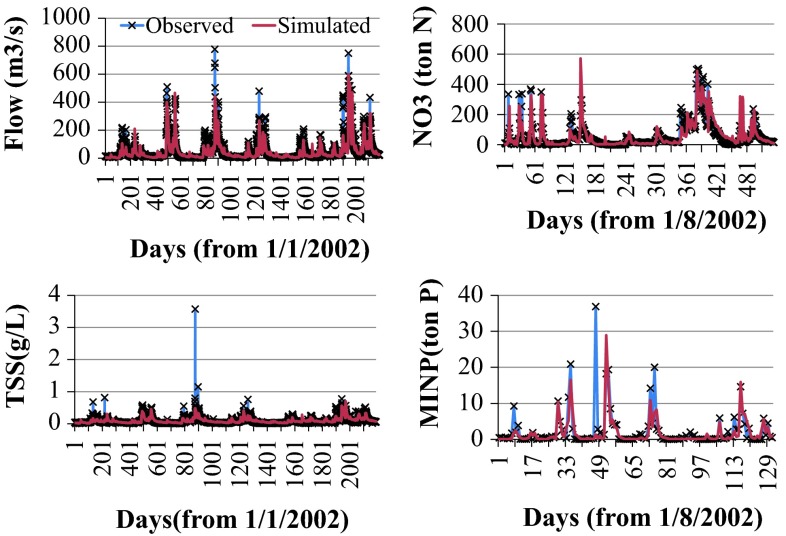
Time serious of simulated and observed daily flow (*upper left*), suspended sediment (*lower left*), nitrate load (*upper right*), and mineral phosphorous (*lower right*) data during calibration period (2002–2007) for RRW (observed NO3 and MINP data were not continuous in the time series)

**Fig. 10:**
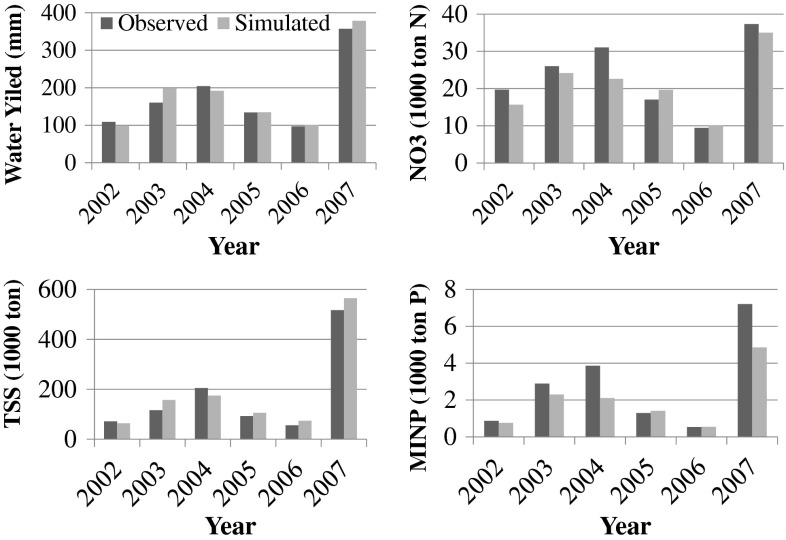
Time serious of simulated and observed annual flow (*upper left*), suspended sediment (*lower left*), nitrate load (*upper right*), and mineral phosphorous load (*lower right*) data during calibration period (2002–2007) for RRW

**Fig. 11:**
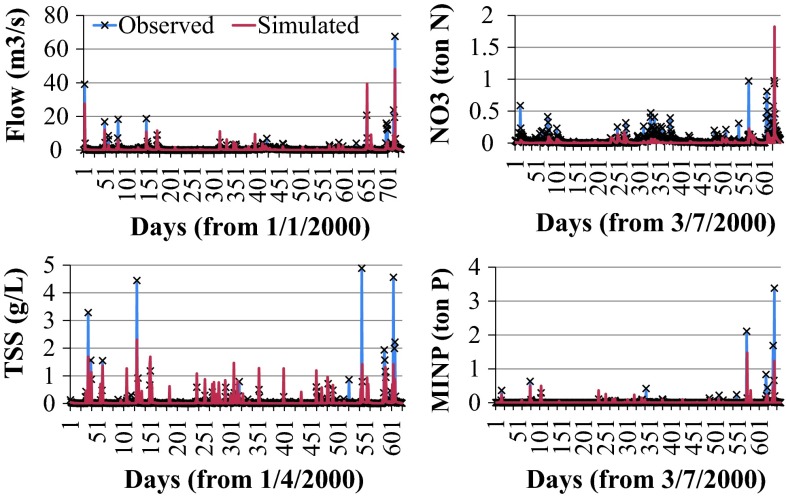
Time serious of simulated and observed daily flow (*upper left*), suspended sediment (*lower left*), nitrate (*upper right*), and mineral phosphorous (*lower right*) load data during calibration period (2000–2001) for BRW (observed TSS, NO3, and MINP data were not continuous in the time series; therefore, LOADEST has been used to interpolate missing daily data)

**Fig. 12:**
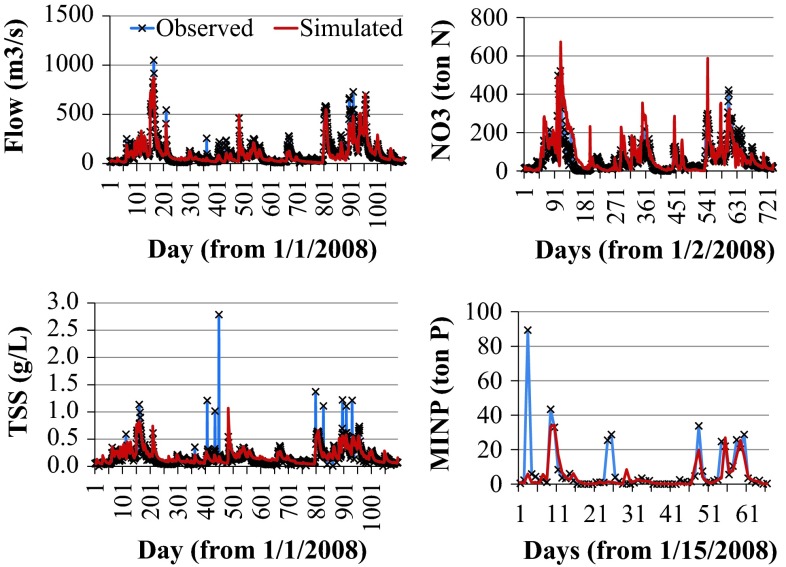
Time serious of simulated and observed daily flow (*upper left*), suspended sediment (*lower left*), nitrate (*upper right*), and mineral phosphorous (*lower right*) load data during validation period (2008–2010) for RRW

**Fig. 13:**
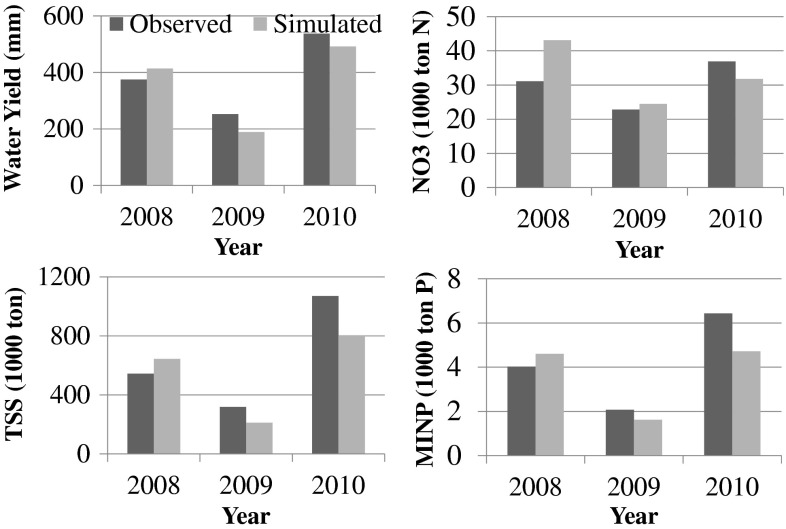
Time serious of simulated and observed annual flow (*upper left*), suspended sediment (*lower left*), nitrate load (*upper right*), and mineral phosphorous load (*lower right*) data during validation period (2008–2010) for RRW

**Fig. 14:**
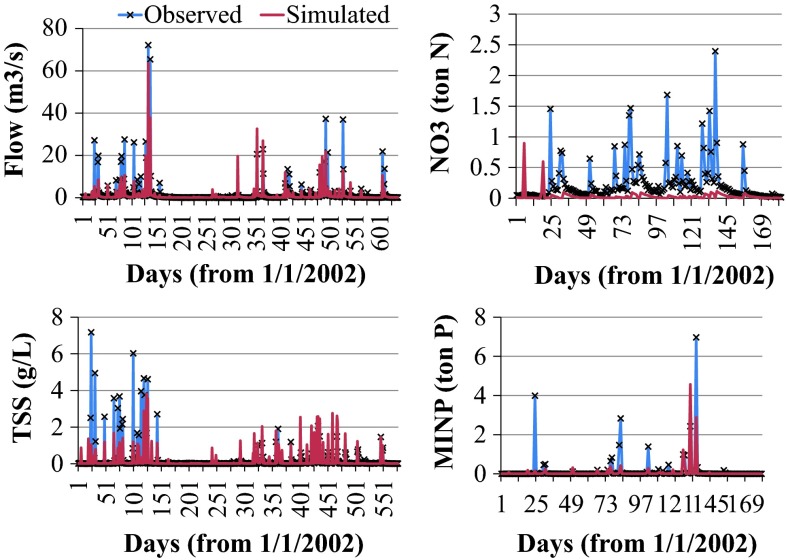
Time serious of simulated and observed daily flow (*upper left*), suspended sediment (*lower left*), nitrate (*upper right*), and mineral phosphorous (*lower right*) data during validation period (2002–2003) for BRW

## Appendix

See Tables 6 and 7. See Figs. 9, 10, 11, 12, 13, and 14.

**Table 6:** SWAT parameters used during SUFI2 automatic calibration and their descriptions

Calibrated SWAT output	SWAT calibration parameter	Description	RRW	BRW
Streamflow (Q)	ALPHA_BF	Baseflow alpha factor (days)	✓	✓
	CANMAX	Maximum canopy storage (mmH_2_O)		✓
	CH_N2	Manning’s “*n*” value for the main channel		✓
	ESCO	Soil evaporation compensation factor	✓	✓
	GW_DELAY	Groundwater delay time (days)	✓	✓
	GW_REVAP	Groundwater revap^a^ coefficient	✓	✓
	MSK_CO1	Calibration coefficient used to control impact of the storage time constant for normal flow		✓
	MSK_CO2	Calibration coefficient used to control impact of the storage time constant fro low flow		✓
	REVAPMN	Threshold depth of water in the shallow aquifer for revap or percolation to the deep aquifer to occur (mm H_2_O)		✓
	SFTMP	Snowfall temperature (°C)	✓	✓
	SMFMN	Melt factor for snow on December 21 (mmH_2_O/°C-day)	✓	✓
	SMFMX	Melt factor for snow on June 21 (mmH_2_O/°C-day)	✓	
	SMTMP	Snow melt base temperature (°C)	✓	✓
	SURLAG	Surface runoff lag coefficient	✓	✓
Sediment (TSS)	CH_COV1	Channel cover factor	✓	✓
	CH_COV2	Channel erodibility factor	✓	✓
	PRF	Peak rate adjustment factor for sediment routing in the main channel	✓	✓
	SPCON	Linear parameter for calculating the maximum amount of sediment that can be reentrained during channel sediment routing	✓	✓
	SPEXP	Exponent parameter for calculating sediment reentrained in channel sediment routing	✓	✓
	USLE_P	USLE equation support practice factor	✓	✓
Nutrient (NO_3_, MINP)	BIOMIX	Biological mixing efficiency	✓	
	ERORGN	Organic N enrichment ratio	✓	✓
	ERORGP	Organic P enrichment ratio	✓	✓
	NPERCO	Nitrogen percolation coefficient	✓	✓
	PHOSKD	Phosphorus soil partitioning coefficient	✓	✓
	PPERCO	Phosphorus percolation coefficient	✓	✓
	PSP	Phosphorus sorption coefficient	✓	✓
	RSDCO	Residue decomposition coefficient	✓	
	SHALLST_N	Initial concentration of NO_3_ in shallow aquifer (mg N/L or ppm)	✓	✓
	SOL_NO_3_	Initial NO_3_ concentration in the soil layer (mg N/kg soil or ppm)	✓	✓

^a^Movement of water from shallow aquifer into overlaying unsaturated layer

**Table 7:** Final ranges and fitted values of SWAT calibration parameters

SWAT calibration parameter	Raccoon watershed					Big creek watershed			
	Final parameter range		Fitted values			Final parameter range		Fitted values	
	Max	Min	Daily	Monthly	Yearly	Max	Min	Daily	Monthly
CN2_AGR	–	–	−0.2^a^			–	–	−0.1^a^	
CN2_PAST	–	–	−0.17^a^			–	–	−0.15^a^	
CN2_FRST	–	–	−0.2^a^			–	–	−0.18^a^	
CN2_URHD	–	–	−0.1^a^			–	–	0.1^a^	
GWQMN	–	–	Default			–	–	1200	
SOL_AWC	–	–	−0.4^a^			–	–	Default	
SOL_K	–	–	0.1^a^			–	–	−0.2^a^	
ANION_EXCL	–	–	0.95			–	–	Default	
ALPHA_BF	0.16	0.25	0.16	0.23	0.24	0.029	0.185	0.073	0.149
CANMAX	–	–	Default	Default	Default	1.4	3.5	1.8	3.4
CH_N2	–	–	Default	Default	Default	0.0010	0.0607	0.0137	0.0135
ESCO	0.90	1.00	0.99	1.00	1.00	0.07	0.67	0.11	0.27
GW_DELAY	66	76	72	68	66	37	48	46	39
GW_REVAP	0.018	0.122	0.110	0.066	0.024	0.161	0.276	0.259	0.172
MSK_CO1	–	–	Default	Default	Default	0.0046	0.0289	0.0115	0.015
MSK_CO2	–	–	Default	Default	Default	0.0037	0.0067	0.004	0.0057
REVAPMN	–	–	Default	Default	Default	25	29	27	27
SFTMP	−2.0	2.0	−0.4	0.5	−1.1	1.4	2.6	2.1	2.1
SMFMN	2.6	3.6	2.8	3.4	2.8	0.03	1.37	0.85	0.53
SMFMX	5.3	6.0	5.9	5.6	5.7	–	–	Default	Default
SMTMP	2.0	3.7	3.5	2.3	2.7	2.3	3.3	3	2.7
SURLAG	0.14	0.47	0.17	0.45	0.45	8	14	13	10
CH_COV1	0.14	0.18	0.17	0.16	0.17	0.21	0.43	0.3	0.31
CH_COV2	0.81	0.91	0.84	0.87	0.91	0.41	0.84	0.79	0.49
PRF	0.23	0.27	0.24	0.25	0.24	0.1	0.32	0.22	0.17
SPCON	0.0013	0.0024	0.0023	0.0019	0.0020	0.0017	0.0049	0.0028	0.0046
SPEXP	2.1	2.6	2.5	2.2	2.4	1.4	1.8	1.7	1.5
USLE_P	0.50	0.77	0.53	0.74	0.76	0.12	0.39	0.19	0.31
BIOMIX	0.38	0.46	0.44	0.43	0.39	–	–	Default	Default
ERORGN	4.1	4.4	4.3	4.2	4.3	2.3	3.2	2.4	3
NPERCO	0.42	0.52	0.51	0.42	0.43	0.17	0.55	0.2	0.47
RSDCO	0.070	0.076	0.072	0.072	0.073	–	–	Default	Default
SHALLST_N	249	676	343	586	451	25	117	99	80
SOL_NO3	38	41	38	38	41	40	54	51	45
ERORGP	1.9	2.4	2.0	2.3	2.0	0.02	0.73	0.27	0.58
PHOSKD	67	88	73	80	74	198	227	217	204
PPERCO	10	17	15	15	16	10	13	12	12
PSP	0.030	0.140	0.140	0.080	0.031	0.034	0.252	0.096	0.1

*AGR* agricultural land, *PAST* pasture, *FRST* mixed forest, *URHD* urban (developed)

^a^Parameter value is multiplied by (1 + *a* given value). For example if CN2_AGR = 78 initially the calibrated CN2_AGR value will be (1 + (−0.2)) × 78 = 0.8 × 78 = 62.4
